# Bread by Weight

**Published:** 1858-02

**Authors:** 


### BREAD BY WEIGHT.

It has been proposed to compel the New York bakers to sell their bread by weight. It is better to have half a pound of bread that is *light* and wholesome, than to have two pounds of sodden bread, heavy as lead almost, and as indigestible as sole leather. Bread is one of the few things which it is best to buy by the bulk, for the lighter it is the better; and the lighter it is, the larger it is. "*Big, and nothing in it*," we heard a little boy exclaim, with considerable indignation, as he brought out a penny mutton-pie, from an eating-house in London. At first sight, we thought it was about as much as a little child could well eat at one time; but on breaking it open, a small mouthful of meat was perceptible in an apartment as large as the Mammoth Cave in Kentucky—*be the same more or less.* No wonder the little fellow objurgated so vehemently. But what there was of it was good; and so it is with nice-looking "French loaves," which are served to New Yorkers, at six cents a piece, weighing (as we have tested ourselves, when, for a change from our old-fashioned yeast-rising, we fancy one), just three quarters of a pound. No baker in New York uses over six dollar flour per bbl., this middle of January, 1858. Let us figure a little. According to the statement of the United States Army Quarter-Master, one barrel of flour makes about two hundred and sixty-five pounds of bread, or three hundred and fifty-three "French" loaves of bread, which, at six and a quarter cents each, amounts to the sum of twenty-two dollars and six cents, for six dollars' worth of flour; and, as there are just fifty pounds of water in the bread which a barrel of flour makes, bakers sell water, which costs nothing, at eight cents a pound. But why do city people give twenty-two dollars for the bread of a barrel of flour, when, if they made it at home, it would cost but a third of that? Simply because not one cook in fifty will acknowledge an ability to bake bread. They will advertise themselves as experts in that line; but when they are once domiciled with you, they spoil the bread on purpose, knowing that the result will be, that they will get clear of that part of a cook's duties; for it is well known, that New York housekeepers put up with numberless impositions, rather than "make a change." Biddy knows that, and takes advantage of it. But there are a few wives in this city, whose mothers raised them to know and do something practical, and who have too much sterling principle to allow their husbands' money to be wasted. You never see a baker's cart drive up to their door, and another sight you never saw—you never saw these women come out of a New York boarding-school. What fools are we! Three-fourths of us are rearing our daughters, on the assumption that they are to marry men with princely incomes. No wonder is it—and right is it that it should be so—no wonder is it, that throughout this whole land, the rule is, that fortunes should change hands every other generation. The morning of our rich men's daughters rises in sunshine and splendor; but the evening goes out in pining penury, and in withering neglect.

Let us not close this article, without having something in it which is tangible and practical. It is stated, that twenty-seven pounds of good bread have been made of fourteen pounds of flour, and half a pound of rice, as follows:

Tie up the rice in a thick linen bag, allowing it ample room to swell.

Boil it three or four hours, until it becomes a smooth paste.

Mix this, while warm, with the flour, adding the usual quantity of yeast and salt; allow the dough to rise near the fire, divide it into loaves, and bake it.

It is said, that the flour thus treated, will yield fifty per cent more bread than by the ordinary method.

